# From action representation to action execution: exploring the links between cognitive and biomechanical levels of motor control

**DOI:** 10.3389/fncom.2013.00127

**Published:** 2013-09-18

**Authors:** William M. Land, Dima Volchenkov, Bettina E. Bläsing, Thomas Schack

**Affiliations:** ^1^Department of Neurocognition and Action Biomechanics, Bielefeld UniversityBielefeld, Germany; ^2^Research Institute for Cognition and Robotics (CoR-Lab), Bielefeld UniversityBielefeld, Germany; ^3^Cognitive Interaction Technology - Center of Excellence (CITEC), Bielefeld UniversityBielefeld, Germany; ^4^Department of Physics, Bielefeld UniversityBielefeld, Germany

**Keywords:** mental representation, basic action concepts, kinematic structure, spatio-temporal kinematic decomposition of movement, structure dimensional analysis—motorics, hierarchical motor control, memory structure

## Abstract

Along with superior performance, research indicates that expertise is associated with a number of mediating cognitive adaptations. To this extent, extensive practice is associated with the development of general and task-specific mental representations, which play an important role in the organization and control of action. Recently, new experimental methods have been developed, which allow for investigating the organization and structure of these representations, along with the functional structure of the movement kinematics. In the current article, we present a new approach for examining the overlap between skill representations and motor output. In doing so, we first present an architecture model, which addresses links between biomechanical and cognitive levels of motor control. Next, we review the state of the art in assessing memory structures underlying complex action. Following we present a new spatio-temporal decomposition method for illuminating the functional structure of movement kinematics, and finally, we apply these methods to investigate the overlap between the structure of motor representations in memory and their corresponding kinematic structures. Our aim is to understand the extent to which the output at a kinematic level is governed by representations at a cognitive level of motor control.

## Introduction

Research on expertise in sports has shown that skilled performance is based not only on physical ability, but equally on task-specific cognitive competences. During extensive practice, relevant mental representations are formed, adapted, and re-organized in such a way that flawless performance is progressively facilitated, based on increasing order formation in the athlete's long-term memory. According to the perceptual-cognitive perspective, actions are planned and performed on the basis of structured cognitive representations of action effects in motor memory (Hommel et al., 2001; Mechsner et al., 2001; Schack and Mechsner, 2006; Hoffmann et al., 2007; Shin et al., 2010). Furthermore, because these representations govern the tuning of motor commands and muscular activity patterns, skillful coordination occurs when appropriate mental representations of the motor task and action goals are constructed (Schack and Ritter, in press). In order to illustrate how these processes can be conceptualized and explored empirically, we will present studies that investigated the organization of task-related cognitive structures, and the way these structures correspond to functional components of skilled motor performance. Additionally, we will present a new empirical approach for linking these mental structures to the structures observed within the movement kinematics. Before we turn to the methodological aspects of these studies, we will first present the underlying theoretical conceptualization of the cognitive architecture of human motor action, beginning with the concept of mental representations, which is fundamental to this approach.

## Mental representations of human motor action

The idea that cognitive representations play an important role in motor control is reminiscent of classical ideas in psychology, such as the “ideomotor” approach adopted by Lotze (1852) and James (1890) in the 19th century or the model-theory studies of the construction of movement presented by Bernstein (1947) in the middle of the 20th century. James wrote for instance 1890 in his now seminal work *The Principles of Psychology*: “We may …lay it down for certain that every representation of a movement awakens in some degree the actual movement which is its object” (p. 526). Recently, the term mental representation has been widely used in a large variety of disciplines, often with rather diverse content. Gilbert and Wilson (2007) have stated: “the mental representation of a past event is a memory, the mental representation of a present event is a perception, and the mental representation of a future event is a plan.” Even though this definition sounds viable, it might not be sufficient for our purposes. Mental representations were first discussed in the philosophy of language, referring mainly to linguistic representations. Later, the issue was adapted by other disciplines such as philosophy of mind and psychology, and various theories have been formulated to describe the nature of mental representations. From these theoretical perspectives, the functionalistic one seems most relevant in our context, as it states that mental representations predominantly play a functional role for the cognitive system. According to this perspective, the function of mental representations is to make situations and objects cognitively available that are otherwise physically unavailable—in this respect, they are the only way to make non-actual situations and objects available for thinking and acting (Vosgerau, 2009).

Several authors have reflected upon the nature of mental representations of actions (e.g., Rosenbaum et al., 2001), and it has been argued that even mental representations of static objects are dynamic in nature, as they are derived from and based on dynamical action representations, which are evolutionarily more relevant for controlling behavior than representations of static scenes or objects (Freyd, 1987). In the context of the studies presented in the following, we refer to mental representations in terms of states of mind that correspond to experiences, and to the physical reality of objects and movements. Such internal representations arise from exposure to sensory stimuli, are multimodal, and refer to objects or events that we perceive in our environment via the processes of perception and processing in our brain (Barsalou, 2008). Mental representations occur on different levels and, due to the nature of our nervous system, can be independent of the actual presence of the object that they refer to in the world. Our ability to store such representations is the basis of our ability not only to learn, but also to make plans and predictions regarding what will happen in the future. Wilson (2002) points out that our cognitive apparatus can even construct mental representations of situations that we have never experienced, purely on the basis of linguistic input. Mental representations thereby play a central role in the control and organization of actions, serving as “organizers of activity” (Steels, 2003). In cognitive systems, internal representations co-evolve together with corresponding actions and become vehicles for higher mental functions, such as thinking and planning (Steels, 2003). As a consequence, these representations stay closely connected to the actions they serve (Glenberg, 1997), resembling them most crucially in terms of structural similarity (Johnson-Laird, 1989). Mental representations are considered vital for learning complex movements and movement sequences, for refining and adapting learned movements to the requirements of actual situations, and for automatizing movement patterns on an expert level. Expert performance in sports is typically characterized by a high degree of control and a sense of clarity, which can arise based on regularities in the mental representation that allow for relieving cognitive load, or, as Wilson (2002) has put it, for circumventing the “representational bottle neck.”

## Representations as a basis for action control

Current perspectives in cognitive psychology suggest that actions are represented in terms of their anticipated perceptual effects (e.g., Prinz, 1997; Hommel et al., 2001; Knuf et al., 2001). Interestingly, these perspectives resonate with the earlier ideas of Bernstein (1996a,b, 1967) regarding the construction and control of movements. Prior to the current perspectives, Bernstein had already pointed toward the large number of degrees of freedom in the human motor system, the need for continuous processing of sensory feedback to control this highly redundant system, and the importance of the anticipation of movement effects for movement organization. Bernstein (1996a,b, 1967) proposed a model of the construction of movements according to which different organizational (and evolutionary) levels interact to generate and control different types of movement. These levels are thought to interact not simply in a fixed hierarchical manner, but their mode of interaction and hierarchical organization depends on the type of movement task and the level of expertise of the performer. Bernstein's model claims that movements are constructed on the basis of five levels described as (1) paleokinetic regulation (regulation of muscular tonus and basic postures, including tonic reflexes), (2) synergies (dynamical stability of movement, rhythmic and cyclic movement patterns), (3) movement in space (spatial orientation and object manipulation), and (4) action (volitional control of movements, object-related action, focus of attention), and (5) symbol coordination (symbolic action control, speech).

Bernstein's model reflects the general idea that movement control is based on representations, which serve intentional movement planning, and that these representations reflect the functional movement structure. Alongside Bernstein's approach to the construction of action, there have been several formulations of the idea that movement control is constructed hierarchically. The model we propose here provides a comprehensive account for the way complex movements are controlled, stemming from the volitional initiation of the action to the lowest level of motor control. Thus, this model acts to provide the relevant framework for a connection between mental representations and motor output. Specifically, the model proposed views the functional construction of actions (Schack, 2004; Schack and Ritter, 2009; Maycock et al., 2010) on the basis of a reciprocal assignment of performance-oriented regulation levels and representational levels (see Table 1). These levels differ according to their central tasks on the regulation and representation levels. Each level is assumed to be functionally autonomous.

**Table 1:** **Levels of motor action (modified from Schack, 2004; Schack and Ritter, 2009)**.

**Code**	**Level**	**Main function**	**Subfunction**	**Tools**
IV	Mental control	Regulation	Volitional initiation control strategies	Symbols; strategies
III	Mental representation	Representation	Effect-oriented adjustment	Basic action concepts
II	Sensorimotor representation	Representation	Spatial-temporal adjustment	Perceptual representation Internal models
I	Sensorimotor control	Regulation	Automatization	Motor primitives basic reflexes

Both control levels, the *level of sensorimotor control* (I) and the *level of mental control* (IV), serve the main function of regulation, whereas the *level of sensorimotor representation* (II) and *level of mental representation* (III) are representational, and are closely connected to the two regulation levels. Levels I and II could be understood as responsible for the functional manipulation of objects and events, whereas levels III and IV can be assigned a more distal focus on objects and events. All levels are connected and interact with each other, but are functionally autonomous.

The *level of sensorimotor control* (I) is based on movement primitives and directly linked to the environment. It is induced perceptually, built on functional units composed of perceptual effect representations, afferent feedback, and effectors. The essential invariant, or set value, of such functional units is the representation of the movement effect within the framework of the action. The system is broadly autonomous, and automatisms emerge when the level of sensorimotor control possesses sufficient correction mechanisms to ensure the stable attainment of the intended effect. Studies of patients with impaired motility showed that the execution of movements can best be realized via anticipated sensory effects, and that such direct sensory effects are the crucial invariant of movement control (e.g., Van der Weel et al., 1991).

Modality-specific information representing the effects of the particular movement is stored on the *level of sensorimotor representation* (II). The relevant sensory modalities might change as a function of the level of expertise in the learning process and as a function of the task context. Grasping movements, for instance, are associated with kinesthetic, tactile, visual, and (in part) auditory feedback. This involves the representation of perceptual patterns of exteroceptive and proprioceptive effects that result from particular movements and refer back to the action goal. During the first steps of learning a novel complex motor action in sports, visual information is often used to monitor body posture and movement timing. In later stages of the learning process, proprioceptive information gains increased meaning, and aspects related to body position and timing are no longer needed to be monitored consciously. During the learning process, movement automatization is characterized by increasingly adequate correction mechanisms between levels I and II.

The *level of mental representation* (III) predominantly forms a cognitive workbench for the *level of mental control* (IV). The *level of mental representation* is organized conceptually, and is responsible for transforming the anticipated action effects into movement programs that sufficiently bring about desired outcomes. According to Bernstein (1967), an action is a structure subdivided into details, and action organization therefore has to possess a working model of this structure, containing the topology and spatiotemporal effects of the action. Mental representations of movement structures that serve this purpose are located within the *level of mental representation (III)*, and are based on the conceptual building blocks of action Basic Action Concepts (BACs) that will be described in the following section.

The *level of mental control* (IV) is induced intentionally and is relevant for the anticipation of effects. Movements are planned, controlled, and performed with reference to the anticipated effects, or intended goal postures (e.g., Rosenbaum and Jorgensen, 1992; Rosenbaum et al., 1992; Kunde and Weigelt, 2005). Findings from such studies suggest the existence of a mental model of the action, including its outcome, to which all control processes can be related. Level IV comprises functional components of volition or mental control, such as the coding of intended effects into action goals. Specifically in the context of sports, instructions and self-applied strategies for focusing attention and stabilizing performance are important aspects of mental control on this level.

### Representation units in motor action

Perceptual-cognitive approaches propose that motor actions are formed by cognitive representations of target objects, movement characteristics, movement goals, and the anticipation of potential disturbances. Movements can be understood as a serial and functional order of goal-related body postures (Rosenbaum et al., 2001) and their transitional states. Furthermore, the link between movements and perceptual effects is bi-directional and based on information that is typically stored in a hierarchical fashion in long-term memory.

Based on Schack's Cognitive Architecture model (see Table 1), complex movements can be conceptualized as a network of sensorimotor information. The better the order formation in memory, the more easily information can be accessed and retrieved. This leads to improved motor performance, which reduces the amount of attention and concentration required for successful performance. The nodes within this network contain functional subunits, or building blocks, that relate to motor actions and associated perceptual and semantic content. These building blocks, termed BACs can be understood as representational units in memory that are functionally connected to perceptual events; or as functional units for the control of actions at the level of mental representation, linking goals at the level of mental control to perceptual effects of movements. Such BACs are activated by representations of starting conditions and deactivated by effect representations, both at the perceptual level.

Underlying neurocogntitive theories state that actions are represented in functional terms as a combination of action execution and the intended or observed effect, or movement goal (Prinz, 1997; Hommel et al., 2001; Knuf et al., 2001; Koch et al., 2004). BACs can be regarded as cognitive tools for the execution of actions such as complex movement tasks in sports (see Schack, 2004). Within these tasks, BACs serve the purpose of reducing the cognitive effort necessary for controlling the action. The same applies to actions performed in everyday life, as their successful execution often depends on experience and thereby requires a level of expertise the performer is hardly aware of.

Altogether BACs can be viewed as the mental counterparts of functionally relevant elementary components or transitional states of complex movements. They are characterized by recognizable perceptual features. They can be described verbally as well as pictorially, and can often be labeled with a linguistic marker. “Turning the head” or “bending the knees” might be examples of such BACs in the case of, say, a complex floor exercise. As mentioned above, each individual BAC is characterized by a set of closely interconnected sensory and functional features. For example, a BAC in tennis like “*whole body stretch motion*” is functionally related to providing energy to the ball, transforming tension into swing, stretching but remaining stable, and the like. Afferent sensory features of the corresponding submovement that allow monitoring of the initial conditions are bended knees, tilted shoulder axis, and body weight on the left foot. Re-afferent sensory features that allow monitoring of whether the functional demands of the submovements have been addressed successfully are muscles stretched and under tension, proprioceptive and, finally, perhaps visual perception of the swinging arm and ball in view.

BACs are stored at a basic level of representation and are investigated and defined by experimental methods (like reaction time-measurement) or with the help of biomechanical methods. Altogether the methods are used to learn about the basic body postures of a particular movement and their mental counterparts in memory. The number of BACs that can be assigned to a given movement task depends on the complexity of the task, on the way it has been learned and trained, and on the level of expertise of the addressee. It is hardly possible to define BACs without the extensive feedback and cooperation of persons who master the task with varying levels of expertise, taking into account their different types of knowledge. Consequently, it is important to take the experience of teachers into account, and to also look at the way the task is actively structured during learning and training, as concepts that emerge during training are likely to remain intact as scaffolding in long-term memory. During the experimental procedure, BACs can be represented as pictures or verbal labels that are meaningful to the participants in order to trigger movement-related long-term memory content. Pictures and verbal labels differ slightly in the way they address mental representations. Presenting an action in pictures instead of words commonly allows for a higher temporal resolution, however, dynamical cues cannot be represented in static pictures unless the stimuli are augmented by verbal terms or symbols (e.g., arrows). Furthermore, pictures represent very short time segments that have a clear temporal order within the action, whereas verbal terms can relate to longer-lasting and synchronous partial actions.

## Measuring mental representations

In principle, there are two methodological approaches to the experimental study of mental representation structures: to determine them from response behavior or to determine them from reaction times. Whereas the first approach has been used for the study of order formation in LTM, the second approach should be used only to ascertain chunk structures in working memory. Schacks architecture model proposes that not only the LTM structure of mental representations but also the exploitation of working-memory capacity serve a notable function in the organization of movement acts. It assumes that working memory forms a unit that is structurally and functionally distinct from LTM. A particular interesting method to measure structures of mental representation in LTM, the so called Structure Dimensional Analysis (SDA) method has been originally developed by Lander and Lange (1996) in cognitive psychology for ascertaining relational structures in a given set of concepts, and adapted by Schack (2001) for analyzing representations of movements (Structure Dimensional Analysis—Motorics, SDA-M). This experimental approach has been documented in several contributions (Schack, 2004; Schack and Mechsner, 2006; Hodges et al., 2007; Schack and Hackfort, 2007; Schack, 2012). Importantly, the method does not ask the participants to give explicit statements regarding their representational structures, but rather reveals this structure by means of knowledge-based decisions in an experimental setting. Altogether, the SDA-M consists of four steps: First, a special splitting procedure requires one to subjectively differentiate whether or not a given BAC is “functionally close” to another, or not. A randomly selected BAC is presented as the standard unit, or anchor, and all other BACs are displayed below the anchor in a randomly ordered list. One after another, each BAC is subjectively compared for similarity to the anchor. Thereby, the list of BACs is split into two subsets, a positive (“close”) and a negative (“not close”) set, which are then repeatedly submitted to the same procedure, until every BAC has been compared to every other. Based on the participants' decisions, the program sums the positive and negative subsets separately and delivers an Euclidian distance scaling between the items (BACs). Second in the process, a hierarchical cluster analysis is used to transform the set of items into a dendrogram.

Third, a dimensioning of the cluster solutions is performed through a factor analysis linked to a specific cluster-oriented rotation process, resulting in a factor matrix classified by clusters. Finally, because the cluster solutions can differ both between and within individuals, a within- and between-group comparison of the cluster solutions is performed using a structural invariance measure lambda to determine their structural invariance (Lander, 1991; Schack, 2010). The structural invariance measure is determined based on three defined values: the number of constructed clusters of the pair-wise cluster solutions, the number of items within the constructed clusters, and the average quantities of the constructed clusters. The lambda value is calculated as the square root of the product of two factors; one factor being the weighted arithmetic mean of the relative average quantity of the constructed clusters, the other one being the proportional number of clusters in the compared cluster solutions.

SDA-M can be applied in two alternative modes, a direct and an indirect scaling mode. In the direct scaling mode, participants make direct judgments about the functional equivalence of pairs of BACs (BAC × BAC: pairs of BACs are judged as closely or not closely related to each other). In the indirect scaling mode, decisions concerning the functional relationship of BACs are made on the basis of features (e.g., spatial, temporal or force parameters of a given movement) that are assigned to the BACs, with the BACs serving only as anchors, and features being judged as belonging or not belonging to the anchor in the context action (BAC × features: features are judged as closely or not closely related to anchor BACs). To determine classification probabilities of features in relation to BACs, the initial *z*-matrix is transformed into a probability matrix (*p*-matrix), consisting of *p*-values that indicate the classification probabilities of features to individual BACs belonging to clusters. Both modes of the SDA-M method include a hierarchical cluster analysis that reveals clusters of BACs (step 2); the difference is that in the indirect scaling mode the features are predefined, whereas in the direct scaling mode the concept dimensions can be accessed via a factor analysis (step 3).

## Previous studies investigating mental representation structures

In the past, the SDA-M method has been used to study differences between groups who vary in their experience with cognitive or motor tasks. In the following, examples of such studies will be given to demonstrate the broad spectrum of potential applications of the Cognitive Architecture approach.

Schack and Mechsner (2006) applied the SDA-M method to investigate participants’ mental representations of the tennis serve by comparing the structures of high-ranking tennis players, low-ranking players, and novices. With the help of tennis experts and coaches, 11 BACs were defined for the tennis serve in relation to the functional movement structure that can be derived on the basis of biomechanical movement parameters. According to this structure, the tennis serve consists of a pre-activation phase that serves to build up tension energy, the strike as the main functional phase during which the energy is conveyed to the ball, and the final swing during which the racket is decelerated and the body is brought back into a stable position. The results of the study showed that the mean group cluster solution of the high-ranking players corresponded to the functional movement structure, reflecting the three functional phases. The low-ranking players' cluster solution combined the strike and the final swing into one cluster that was differentiated from the pre-activation phase. The novices' solution did not contain any clusters, reflecting a lack of functional order formation within this group. Thus, the authors found that the mental representation structure of the experts were well matched to the functional and biomechanical demands of the task, whereas, the low-ranking and novice players' representations were less hierarchically organized and not matched to the biomechanical demands of the task.

Similarly, Bläsing et al. (2009) applied the SDA-M method to compare the mental representations of classical dancers varying in expertise level and dance novices. Mental representations of two well-defined movements from the classical dance repertoire, the *pirouette en dehors* and a small jump called *petit pas assemblé*, were investigated in professional ballet dancers, advanced ballet amateurs, amateur beginners, and sport students without any dance training experience. The results for the *pirouette* revealed that the mean cluster solutions of both groups of professional dancers and advanced amateurs corresponded to the functional phases, with only minor differences. The beginners' cluster solution differed largely from the others and did not show much alignment with the functional phases, and the novices hardly formed any relevant clusters. For the *pas assemblé*, no difference was observed between the two groups of amateurs. Amateurs and novices formed clusters that included all BACs and were similar to the clusters formed by the experts, only the experts' cluster solution reflected the dynamical initiation of the jump, corresponding to the functional phases.

In a follow-up study, the SDA-M method was applied in indirect scaling mode (BAC × feature), in which the BACs appear only as anchors, and features are sorted in relation to these anchors (Bläsing and Schack, 2012). The same movements were used, and the BACs defined for the first study were presented as anchors. Spatial direction labels as features were related to these anchors, and the participants (who had already taken part in the previous study, Bläsing et al., 2009) were instructed to answer positively for spatial directions which they associated with a given BAC within an egocentric reference frame, according to their own motor imagery of the movement. Results showed that for the *pirouette*, only the professional dancers' mean cluster solution contained one cluster that clearly corresponded to the main functional phase and associated this phase with relevant directions. The amateurs' mean cluster solution did not result in any functional clusters, and the novices' did not contain any clusters at all. For the *assemblé*, professionals' and amateurs' cluster solutions associated the main functional phase with relevant spatial directions, whereas in the novices' cluster solution did not correspond to the functional movement parameters.

The SDA-M method has not only been applied to compare task-related cognitive representations of experts to those of novices, but also to monitor differences between developmental stages. In a study on anticipatory motor planning in children, Stöckel et al. (2012), investigated the end-state-comfort (ESC) effect in children aged 7–9 years. Additionally, the children took part in an SDA-M task with pictures of a hand grasping common objects in different ways. Only the 9-years old children produced a cluster solution that separated uncomfortable from comfortable grasps. In combination with the results of the ESC experiment, the study showed that 9-years old children had more distinct representations of comfortable and uncomfortable grasp postures and a better ability to plan movements to end in comfortable postures compared to younger children. Furthermore, both abilities were found to be related, as children who clustered by grasp comfort also showed the ESC effect, whereas children who did not cluster by grasp comfort performed less consistently in the ESC task, suggesting that cognitive representations of grasp postures are crucial for manual posture and action planning.

In the presented studies, the SDA-M method was applied to investigate general differences in cognitive skill representation between participants of varying levels of expertise or developmental stages. For this purpose, mean cluster solutions of groups of participants were compared, with each group representing a defined level of expertise and cluster solutions being understood as typical for this expertise level. The studies did not, however, pay attention to cluster solutions of individual participants within these groups, nor inter-individual differences in cognitive movement representations. This individual approach was taken by Weigelt et al. (2011), who studied the mental representations of a Judo throwing technique (Uchi-mata) in judoka who were competing on the national team level. The individual cluster solutions of two of the eight participants examined in this study were compared *post-hoc* to the mean group cluster solution, which represented the functional movement phases as expected. The individual cluster solutions differed in details from the mean cluster solution and functional reference structure, reflecting individual preferences and technical differences as well as weaknesses in the judokas' performance. The authors point out that such difference, interpreted with accuracy and care, can reveal subtle flaws in the technical skills of the athlete and can be used by an expert coach to improve and adapt further training.

Comparison between individual cluster solutions and group average cluster solutions has also been used in the context of rehabilitation. Braun et al. (2007) used SDA-M to analyze the mental representations of a common everyday activity, drinking from a cup, in elderly patients recovering from stroke. Sixteen patients 3–26 weeks after their stroke took part in the study as part of their rehabilitation program, along with sixteen matched controls. SDA-M was applied using pictures representing the action sequence, augmented with arrows indicating movement directions if necessary. The results of all participants were regarded individually. The sixteen control subjects produced very similar cluster solutions consisting of two or three clusters corresponding to the functional action phases. The stroke patients' cluster solutions differed largely from those of the control group and from each other and were characterized by a weak functional integration of BACs, ranging from incomplete functional over non-functional clusters to a total absence of clusters. The study showed that SDA-M can be applied as a tool in rehabilitation, even in patients with reduced motor and cognitive capabilities.

Bläsing et al. (2010) used SDA-M to compare individuals with congenitally missing limbs, one of them with congenital phantoms of arms and legs, to different control groups. Instead of BACs, body parts and related activities were used as items in order to evaluate the mental representations of the participants' own bodies. The results revealed that the cluster solution of the individual with congenital phantom limbs, the existence of which had been affirmed in several previous studies using behavioral and neurophysiological methods, differed only slightly from the groups' cluster solutions, showing the same modular structure (Haggard and Wolpert, 2005). In contrast, the result of the individual who had never experienced any phantoms, however, differed largely from this modular structure, but rather reflected the individual's typical use of his body in everyday activities, providing evidence for an action-based influence on the adaptive body representation (Haggard and Wolpert, 2005). The findings from this study suggest that the SDA-M method might provide empirical access to the body schema, which is inextricably linked to the motor system and is constantly involved in the planning and execution of motor actions (de Vignemont, 2010).

The striking differences in representations found between high- and low-level performers support the assumption that motor learning leads to the development of task-specific representations, which play an important role in the control and organization of actions (e.g., Elsner and Hommel, 2001). According to skill acquisition theories (e.g., Fitts and Posner, 1967; Anderson, 1982, 1993, 1995), the cognitive mechanisms governing task performance are refined over the course of learning. To this extent, learning can be viewed as the modification and adaptation of representation structures in long-term memory (Schack, 2004; Schack and Ritter, in press). To directly test this assertion, Frank et al. (2013) investigated the developmental change in mental representation structures over the course of early skill acquisition of a complex motor task. Specifically, the authors employed a longitudinal design in which a group of novices practiced a golf putting task over the course of five training days. Both the change in participants' putting performance as well as the developmental change in the structure of the participants' mental representations were assessed before and after training. Results indicated that along with improved putting proficiency, significant developmental changes emerged within the practice group's mental representation as a result of task practice. These findings support the notion that functional adaptations of mental representations are closely tied to motor learning.

Research in the area of training and feedback has indicated that the type of attentional focus induced by training instructions can significantly impact the quality and rate of skill acquisition. To this extent, instructions that promote an external focus of attention (i.e., attention given to the effects of the movement on the environment) can lead to improved motor learning and retention (e.g., Wulf et al., 1999), reduced working memory demands (e.g., Wulf et al., 2001), reduced susceptibility to performance pressure (e.g., Land and Tenenbaum, 2012), and overall, better outcome performance (e.g., McNevin et al., 2003). Given that the sensory consequences of motor actions are considered an important component within mental representations (e.g., Ford et al., 2007), it appears likely that focusing on the sensory effects of one's movement (i.e., an external focus) during learning may act to facilitate the integration of perceptual effects during the formation of one's mental representation, leading to a more refined representation structure.

To explore this question, Land et al. (submitted) examined the developmental change in participants' representation structures as a function of instruction type. Specifically, novice participants trained on a golf putting task over the course of three training days. For half of the participants, training instructions were given to direct attention to the external effects of their movement (i.e., the roll of the golf ball). For the other half of participants, instructions were given such that attention was directed internally to the execution of the movement (i.e., focus on the swing of the arms). At the conclusion of practice on the third day, results indicated that both training groups displayed improved putting performance along with significant functional changes in their underlying mental representation. However, the performers who were given instructions that directed attention to the sensory consequences of their movement significantly outperformed the group who trained while focusing on skill execution. Additionally, the representation structure of the external learning group was significantly more elaborate and more functionally similar to skilled golfers than those of the internal focus learning group. These findings highlight that the association between movements and their perceptual effects are crucial for learning. To this extent, findings suggest that instructions which emphasize an external focus of attention aid the integration of perceptual effects during the development of mental representations. These findings furthermore give credence to the assertion that sensory consequences of motor actions are an important component within mental representations.

## Investigating the relationship between representations and motor organization

The results of the aforementioned research indicate a clear relationship between mental representations and motor performance. Specifically, these findings support the hypothesis that voluntary actions are planned, executed, and stored in long-term memory by means of reference structures comprised of BACs. Central to this perspective, mental representations are functionally considered to guide the motor organization during the realization of action goals. In this regard, these representations serve as a cognitive reference for the creation of motor patterns. Given that movements are structured and controlled via these representations, an important theoretical advancement would be to identify direct links between mental representations and movement kinematics in the fulfillment of action goals.

In this direction, Schack (2003) investigated the relationship between mental representations of gymnastic somersaults and the underlying movement kinematics amongst gymnasts of varying skill levels. The results indicated significant correlations regarding the space and time of the movement between kinematic parameters and the structural relationship between BACs within the motor representation. For instance, a significant negative correlation was found between the angular velocity of the somersault and the Euclidean distance between two nodes within the representation structure related specifically to the initiation of the twisting motion.

In a similar investigation, Schütz et al. (2009) were able to show that key biomechanical parameters of a table tennis serve could be modeled based on the mental representation structure of the participants. The movement kinematics and the mental representations of nine table tennis experts revealed that movement duration and ball flight parameters were predictable based on the Euclidean distance between select representation nodes. For example, the movement duration of the table tennis strike could be predicted by the representational distance between the individual BACs “move racket backward” and “move racket downward and forward.”

The implications of these two studies indicate that representational structures can be used to predict kinematic parameters of movement for a given task. Specifically, the relationships between local subsets of BACs within a representation structure have been found to be associated with spatial and temporal aspects of movement. However, given that mental representations serve as a reference structure for the unfolding of a movement pattern, it would be important to find a direct link between the structure of the representation and the overall structure of the movement on a more global level. For this to occur, a proper method for decomposing a movement into its structural features must be proposed, which we will do in the following section.

## Spatio-temporal kinematic decomposition of movement

The modern technologies of motion tracking provide researchers with a wealth of kinematic data on the full-body movements of humans, animals, and various robotic platforms. In order to explore this rich data, we have created computationally feasible algorithms for decomposing movements into independent spatio-temporal features directly from the captured kinematic signal. The proposed approach is useful for understanding, interpreting, and modeling complex movements in systems possessing many degrees of freedom, and provides a means for examining the overall structure of a movement.

The following algorithms have been tested on recorded movements from classical ballet and golf, and they allow us to estimate the level of movement expertise, draw the detailed structures of arbitrary complex movements, and automatically classify them into a given repertoire.

From our work on the kinematic analysis of complex full-body movements in classical ballet (see Volchenkov and Bläsing, 2013), we show that a movement tracked by a motion tracking system (MTS) can be understood in terms of a hierarchy of major and minor scales, in which the spatial and temporal components can be separated and studied independently. Based on our Spatio-Temporal Kinematic Decomposition (STKD) method, the major structure of a movement can be assessed. Specifically, the affinity between markers is identified by measuring the distance between them in the largest scale of kinematic signal, and by visualizing the results via a dendrogram. This approach reveals the functional relationship between markers by their geometric proximity. The typical character of movement is featured by the few major scales, while the minor scales determine the individual movement traits and can uniquely disclose the individual level of movement expertise, uneven distribution of the fine motor skills, and the emotional character of an individual (see Volchenkov and Bläsing, 2013 for details). The functional separation of scales can explain why we can perceive movements categorically (for example, as the highly stylized figures of classical ballet).

### STKD procedure

**Figure 1:**
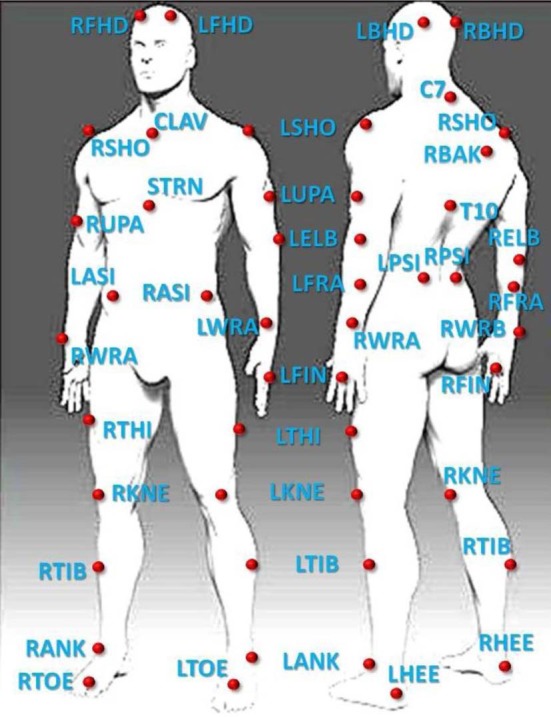
**Location of the passive retro-reflective spherical body markers arranged on the dancer's body in relation to the human skeletal system**.

To track ballet movements (as in Volchenkov and Bläsing, 2013), we used a MTS (Vicon Motion Systems, Inc.) based on 12 high-resolution cameras outfitted with IR optical filters and rings of LED strobe lights streaming data at 200 *fps*; the cameras detected the 3-dimensional spatial positions of passive retro-reflective spherical body markers with millimeter accuracy. Markers were attached to key anatomical locations according to the standard Vicon full-body marker placement protocol (Plug-in Gait) (see Figure 1).

#### Scale decomposition of kinematic data

The MTS delivers positional data for *N* markers, across *T* time frames (*T* >> *N*) at the rate of 200 *fps*, in the form of a rectangular 3*N* × *T* matrix ***M*** = (*x*_1_, *y*_1_, *z*_1_,…, *x_N_*, *y_N_*, *z_N_*), in which the consequent triples of columns, ***x***_*k*_ = (*x*_*kt*1_, …, *x_ktT_*)^**T**^, ***y***_*k*_ = (*y*_*kt*1_, …, *y_ktT_*)^**T**^, ***z***_*k*_ = (*z*_*kt*1_, …, *z*_*ktT*_)^**T**^, represent the Cartesian coordinates of the markers *k* = 1, …, N, at the sequent time frames τ = *t*_1_, …, *t_T_*. The T sign indicates transposition. The data matrix ***M*** is factorized using the *singular value decomposition* (SVD),

(1) $$M=U\Sigma V^{\text{T}}=\sum_{s=1}^{3N}\sigma_{s}u_{s}⊗v_{s}^{\text{T}},$$

where the ⊗ sign stands for the outer product of vectors, *U* is a 3*N* × 3*N* unitary matrix with the columns ***u***_*s*_ representing the left singular vectors of ***M***, *V* is a *T* × 3*N* unitary matrix with the columns ***v***_*s*_ representing the right singular vectors of ***M***, and ∑ is a 3*N* × *N* diagonal matrix of ordered non-negative scale factors (singular values): σ_1_ > σ_2_ ≥ … ≥ σ_3*N*_ > 0. A number of smallest singular values can be equal to zero if the MTS suffers from optical occlusion. Moreover, a number of left and right singular vectors can belong to the same singular value if the matrix ***M*** enjoys an exact spatio-temporal symmetry. However, while processing the actual motion tracking data, we have never encountered multiple singular values. If all singular values of ***M*** are non-degenerate and non-zero, then the factorization (1) is unique, up to simultaneous multiplication of the left and right eigenvectors by the same unit phase factor. The left singular vectors form an orthonormal basis for the spatial arrangement of markers, (***u***_*s*_, ***u***_*s*′_)_*R*3*N*_ = δ_*s, s*′_, with respect to the inner product in _*R*_^3*N*^. The right singular vectors are orthonormal with respect to the inner product in _*R*_^*T*^, (***v***_τ_, ***v***_τ′_)_*RT*_ = δ_τ, τ′_, forming a basis for the temporal sequences of kinematic data. With the use of (1), the kinematic signal ***M*** is decomposed into a weighted, ordered sum of separable matrices σ_*s*_ ***u***_*s*_ ⊗ ***v***^T^_*s*_, in which the information about the spatial arrangement of markers corresponding to the singular value σ_*s*_ is represented by the vector ***u***_*s*_ separately from the vector ***v***_*s*_, giving an account of the temporal evolution. For each non-degenerate singular value, the separable matrix σ_*s*_ ***u***_*s*_ ⊗ ***v***^T^_*s*_ is a rank-one 3*N* × *T* matrix describing a *one-dimensional* mapping of spatial locations of markers to the sequent time frames that correspond to the synchronous motion of all markers (although with variable velocity) along straight lines. Namely, the trajectories of markers specified by the consequent triples of columns **r**^(*s*)^_*k*, (τ)_ = (**x**^(*s*)^_*k*,τ_, **y**^(*s*)^_*k*,τ_, **z**^(*s*)^_*k*,τ_) of the matrix σ_*s*_ ***u***_*s*_ ⊗ ***v***^T^_*s*_ can be described mathematically using a single spatial dimension. Let us denote with ${\vec{\rho }}_{k}^{\left(s\right)}$ the unit vector, tracing the direction of the linear motion of the *k*th marker at the scale *s*_*k*_,

$${\vec{\rho }}_{k}^{\left(s\right)}=\frac{\left(r_{k}(\tau +1)-r_{k}(\tau )\right)}{\left\|r_{k}(\tau +1)-r_{k}(\tau )\right\|},\text{ for any }\tau =t_{1},\ldots t_{T-1}$$

and the amplitude function of the linear motion common for all markers by

$$\gamma_{s}(\tau )=\frac{\left(r_{k}^{\left(s\right)}(\tau ),{\vec{\rho }}_{k}^{\left(s\right)}\right)}{\left(r_{k}^{\left(s\right)}\left(t_{1}\right),{\vec{\rho }}_{k}^{\left(s\right)}\right)},\;\gamma_{s}\left(t_{1}\right)=1$$

Then, the trajectory **r**_*k*_(τ) of the *k*th marker recoded by the MTS can be represented by the ordered sum of linear trajectories,

(2) $$r_{k}(\tau )=\sum_{s\;=\;1}^{3N}{\vec{\rho }}_{k}^{\left(s\right)}\cdot \gamma_{s}(\tau ),\;\tau =t_{1},\ldots ,t_{T}.$$

**Figure 2:**
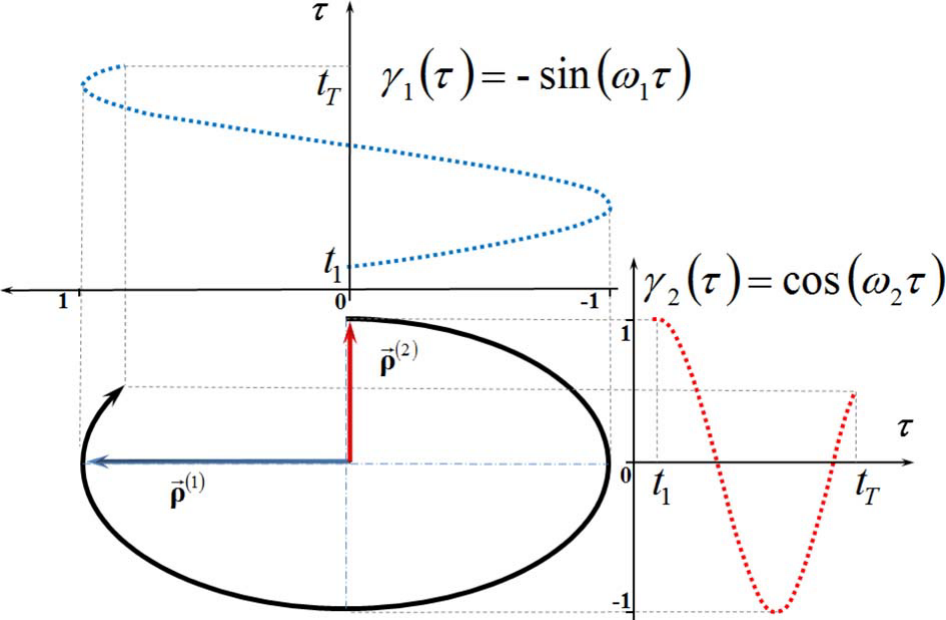
**The scale decomposition of a plane elliptic trajectory segment (reproduced with permission from Volchenkov and Bläsing, 2013)**.

The SVD of trajectories into linear components given by (2) is obvious for the motion of a single marker (see Figure 2) along a planar elliptic trajectory segment. In such a simple case, the components ${\vec{\rho }}^{\left(1\right)}$ and ${\vec{\rho }}^{\left(2\right)}$ are nothing but the major and minor axes of the ellipse. It is clear that the amplitude functions for the bigger and smaller scales of motion are γ_1_(τ) = −sin (ω_1_ τ) and γ_2_ (τ) = −sin (ω_2_ τ), respectively.

Application of SVD in data analysis is similar to the well-known principal component analysis and Fourier analysis (see Jolliffe, 1986). By setting the small singular values to zero, we obtain the minimal set of independent spatio-temporal features, ordered according to the scales of motion, which then approximate the original data with a maximal precision. Namely, for *l* < 3*N*, the 3*N* × *T* matrix *M*^(ℓ)^ = ∑^ℓ^_*s* = 1_σ_*s*_ ***u***_*s*_ ⊗ ***v***^T^_*s*_ renders the best least square approximation to ***M*** of the rank-ℓ, with an error smaller than the first neglected eigenvalue σ_ℓ + 1_. By neglecting the scales σ_*s* > ℓ_ in (1) and consequent recombination of the kinematic signal, we can filter out unsolicited scales of motion (e.g., small scale movements of markers fixed on the clothing instead of the skin, movements of skin and tissues relative to the skeletal system, etc.) Despite certain computational similarity, the method of SVD differs essentially from the latent variable models, such as factor analysis, which use regression modeling techniques to test hypotheses producing error terms. The decomposition (1) does not involve any statistical hypotheses, being a purely descriptive technique.

### Analyzing movement structures

Scale decomposition of tracked movements can be used as a base for the functional alignment of markers. Spatio-temporal relationships between different body parts in evolving movements can be visualized by a dendrogram representing the relative distance between markers on the largest scale of movement through the horizontal branch length. In accordance with (2), a motion can be understood in terms of a hierarchy of scales evolving by γ_*s*_ (τ). The lowest level of this hierarchy corresponds to fast, low-scale movements of markers fixed on the clothing relative to the body, whereas the highest levels encode relatively slow, large-scale movements of the skeletal system. Although a detailed analysis of the functions γ_*s*_ (τ) lies beyond the scope of the present paper, it is worth mentioning that they typically constitute strongly anharmonic oscillations, indicating that the relationship between force and displacement at each movement scale is strongly non-linear.

Being primarily concerned with the movement on its largest scale, we note that its structure is determined in (2) by the spatial arrangement of vectors ${\vec{\rho }}_{k}^{\left(1\right)}$ in association with the markers *k* = 1, …, N. For each marker, the magnitude of the vector $\left\|{\vec{\rho }}_{k}^{\left(1\right)}\right\|\cdot \sum_{\tau =t_{1}}^{t_{T}}\left|\gamma_{s}(\tau )\right|$ can be considered as a relative measure of its mobility on the movement scale σ_*s*_. The degree of affinity between a pair of markers, *k*_1_ and *k*_2_, can be attested on the largest scale of the movement by means of the Euclidean distance between the related vectors,

(3) $$d\left(k_{1},k_{2}\right)=\left\|{\vec{\rho }}_{k_{1}}^{\left(1\right)}-{\vec{\rho }}_{k_{2}}^{\left(1\right)}\right\|$$

It is customary to reproduce the matrices of all-to-all distances in the form of a dendrogram by placing closely-related markers in the same mold. To preserve the structure (3) as much as possible, we use the standard neighbor-joining tree-generating algorithm (Felsenstein, 2004). We search the matrix (3) for the closest markers, and then connect them into a block. Once the markers are connected, they are removed from the distance matrix and replaced by the block connecting them. The neighbor-joining algorithm continues until all *N* markers are connected in a tree, and each branch acquires a length, with length being interpreted as the estimated number of substitutions required to resolve the block. The functional contingency between blocks of markers on the largest scale of the movement is disclosed by their geometric proximity in the resulting dendrogram. In spite of all participants sharing roughly the same anatomy and performing the same movements, the structures of calculated dendrograms can be substantially different in terms of individual movement features and level of movement expertise (Volchenkov and Bläsing, 2013).

### Representing functional alignment of markers in the pirouette en dehors

**Figure 3:**
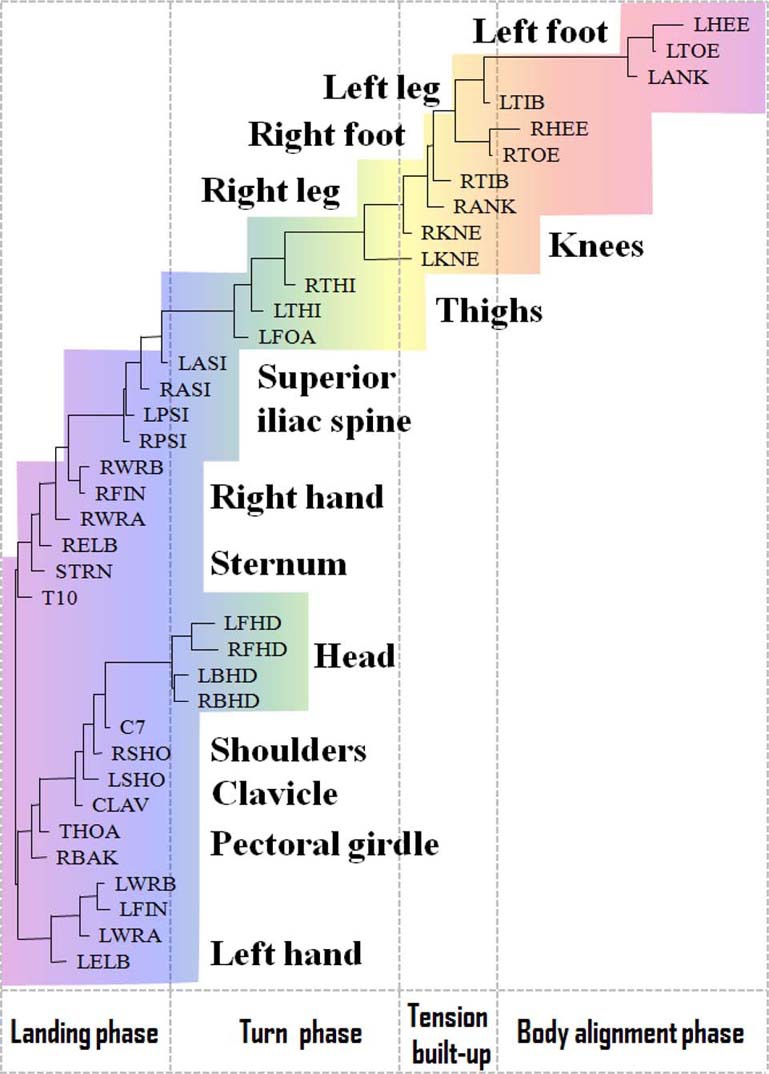
**Functional alignment of markers in *pirouette en dehors* performed by a professional ballet dancer**. The standard Vicon Plug-in Gait marker notations are used (reproduced with permission from Volchenkov and Bläsing, 2013).

**Figure 4:**
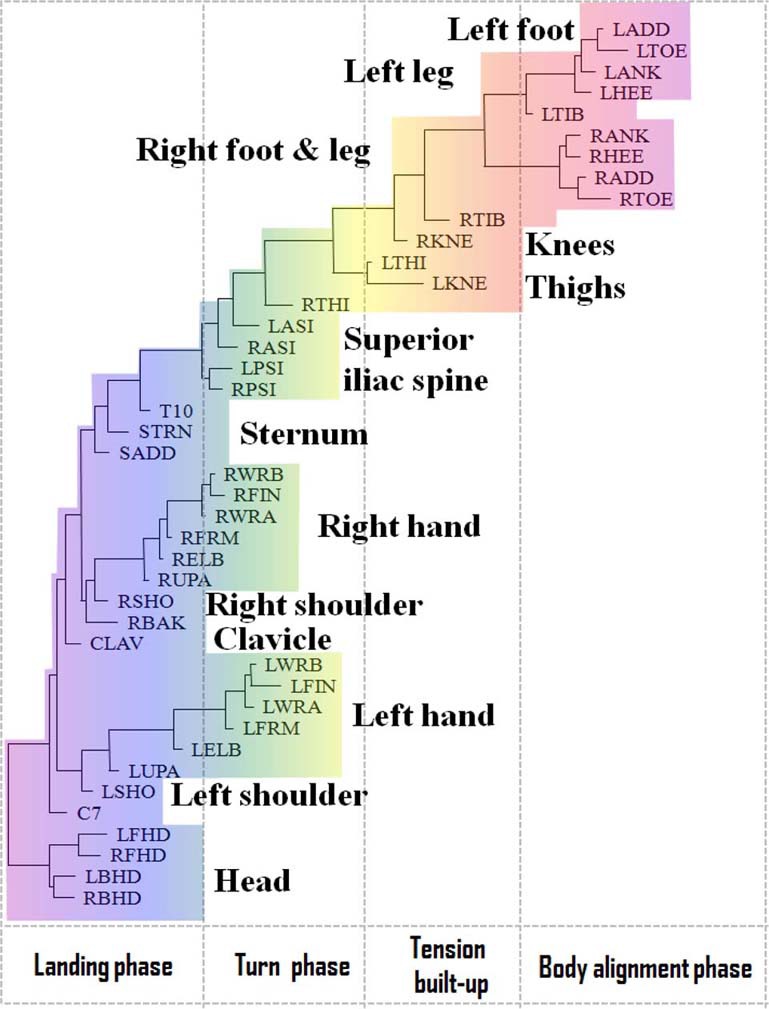
**Functional alignment of markers in *pirouette en dehors* performed by a student**. The standard Vicon Plug-in Gait marker notations are used (reproduced with permission from Volchenkov and Bläsing, 2013).

In Figures 3, 4, we have shown the neighbor-joining dendrograms representing the functional alignment of markers in the *pirouette en dehors* performed by a professional ballet dancer and a novice, respectively. To visualize spatio-temporal relationships between markers on the major scale of recorded movements, we have used the *TreeView* software, which is freely available on the internet (see Page, 1996). We emphasize that the dendrogram shows the functional alignment of markers in the sequential phases of the movement, irrelevant to the actual durations of these phases (see Volchenkov and Bläsing, 2013 for details). The *pirouette en dehors*, a controlled turn away from the supporting leg, is one of the most difficult of all ballet steps that can be executed with single or multiple rotations. The proper turning technique includes a periodic, rapid rotation of the head that serves to fix the dancer's gaze on a single spot, helping her to maintain control over the body (known as *spotting*). This rotational movement requires highly-defined coordination and constant adjustment of the body axis in order to be performed with the required stability and accuracy (Schack, 2001). The rhythmic structure of the *pirouette en dehors* is described by Tarassow (2005) as four measures in 2–4 time, the first two measures containing the preparation, and the second two measures containing the turn and conclusion. According to this approach, the *pirouette en dehors* consists of two parts, the preparation and the actual turning movement. Both of these parts can be dissected again; the preparation can be broken down into two rhythmically-separated sections, whereas the turn segment consists of the actual turning movement and the opening to the front that concludes the turn. The dendrogram shown in Figure 3 discloses the functional structure of the *pirouette en dehors* on the left leg, executed by an expert. On the largest scale of the movement, the pirouette starts (at the upper right corner of the dendrogram) with the function of body alignment by arranging legs in the proper position: the right foot is placed in front of the left foot, both turned outward. The right foot slides to the side (*tendu*, or *dégagé*), which concludes the *body alignment* phase. The spring tension is built up for the turn during the *tension build-up* phase, as the right foot moves back and is placed behind the left one and the knees bend (*plié*). At the beginning of the *turn* phase, both legs push into the ground and the left (supporting) leg adopts *point* or *demi-point* position (on the toes or on the ball of the foot, respectively), while the right knee is bent and the right foot is pulled up to the knee of the supporting leg. During the turn, the head is rapidly whipping around, which helps the dancer to maintain balance. Eventually, in the *landing* phase concluding the turn, the right foot is placed behind the left one, and the knees bend and stretch (*plié*). The arms open, and the arms and torso are used to cease rotation. It is important to mention that each functional phase elicited from the dendrogram shown in Figure 3 can be ascribed directly to the functional phases of the *pirouette en dehors* as defined in Bläsing et al. (2009) via the BACs, the key points within the functional structure of the movement, which are stored in the long-term memory of a dancer.

In contrast to the movement sequence executed by the professional dancer, the movement of a novice performer inappropriately starts simultaneously in both legs, and turning starts prematurely, while straightening the knees (see Figure 4). In the turning phase, the movements are allocated to the superior iliac spine. The head apparently does not play a role until ceasing the movement. Instead, the vigorous hand movements play a major role in maintaining the body's rotation, which is a common mistake among beginners.

## Linking mental and kinematic structures

Given that the STKD procedure identifies the underlying kinematic structure of movements (see Figure 3), we are able to examine whether the organization and structures found within the mental representations share common structural features with those found in the movement kinematics. To provide a first glimpse into this overlap, we examined the movement kinematics and mental representations of a golf swing in 9 participants (*M*_*age*_ = 32.3, *SD*_*age*_ = 10.6, 6 males) of varying skill levels (0–50 years of golf experience). Specifically, movement kinematics of a golf swing were captured using a 3-dimensional MTS (Vicon Motion Systems, Inc.) in which markers were placed on the anatomical landmarks of the body consistent with the standard Vicon full-body Plug-in Gait marker placement protocol. The subsequent marker trajectories for each trial were subjected to the STKD analysis (presented above) to produce a dendrogram of hierarchical couplings of movement trajectories, indicating the kinematic structure of the movement.

Likewise, the mental representation of the golf swing was procured for each participant through the SDA-M analysis (see Schack, 2004, 2012). The SDA-M identified the structural composition of mental representations by revealing the hierarchical and temporal structure of BACs within long-term memory. In order to assess the underlying mental representation on a level consistent with the kinematic structure revealed by the STKD approach (i.e., coupling between body segments), we utilized verbal labels indicating body parts as the representation units for the splitting task, the first step of the SDA-M. These verbal labels consisted of body parts relating to the tracked body segments in the motion-captured kinematic data^1^. Participants were instructed to assess the similarity between each of the body segments in terms of function and motion with respect to the golf swing. The resulting mental representation signified the functional relationship between body segments involved in the golf swing movement in long-term memory. The use of body parts as part of the SDA-M method has been successfully shown to distinguish between individuals with differing body representations in relation to a particular motor task (e.g., Bläsing et al., 2010).

The basis for the comparisons between the representational structure and the kinematic movement structure resides in the Euclidean distance matrices derived from both the STKD and SDA-M analyses. The distance matrix obtained from the SDA-M method is comprised of the Euclidean distances between concepts (body parts) as represented in feature space based on the results of the SDA-M splitting procedure. For the distance matrix obtained from the STKD method, the matrix contains the Euclidean distances between body markers within the major spatio-temporal scale of movement. From these two matrices, the hierarchical structure of the movements were derived, and thusly compared. More specifically, we computed both mean group dendrograms and individual dendrograms for both the mental representation and kinematic data via cluster analysis. Each cluster solution was established by determining an incidental euclidean distance (*d*_*crit*_). Nodes linked together above this critical value were considered unrelated, while BACs linked below this value were considered related. For all cluster analyses conducted, the critical value *d*_*crit*_ = 5.64 was chosen, which reflects an alpha-level of α = 0.001.^2^ Next, the invariance measure λ was calculated to determine the degree of similarity between two cluster solutions. According to Schack (2012), two cluster solutions are invariant (i.e., not significantly different) for λ > 0.68, while two cluster solutions are significantly variant for λ < 0.68. Additionally, the correlation between the distance matrices was examined as a measure of overall relatedness of structural couplings of body parts on a mental and physical level.

**Figure 5:**
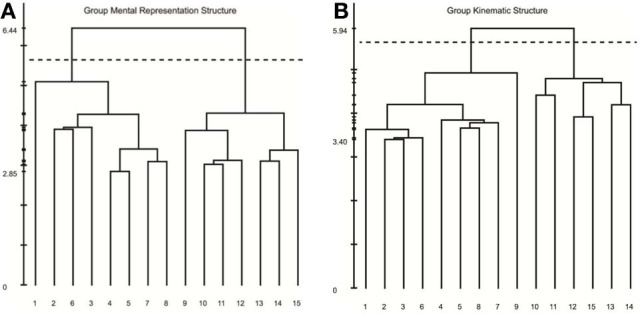
**Mean dendrograms for the mental representation (A) and swing kinematics (B)**. The numbers on the horizontal axis relate to the concept number, the numbers on the vertical axis display Euclidean distances. The lower the link between related concepts, the lower is the Euclidean distance between the corresponding concepts in feature space. The horizontal dotted line marks *d*_*crit*_ value for a given α-level (*d*_*crit*_ = 5.64; α = 0.001): links below this line are considered statistically relevant. Concepts: (1) head, (2) chest, (3) left shoulder, (4) left elbow, (5) left hand, (6) right shoulder, (7) right elbow, (8) right hand, (9) hips, (10) left thigh, (11) left knee, (12) left foot, (13) right thigh, (14) right knee, (15) right foot.

**Figure 6:**
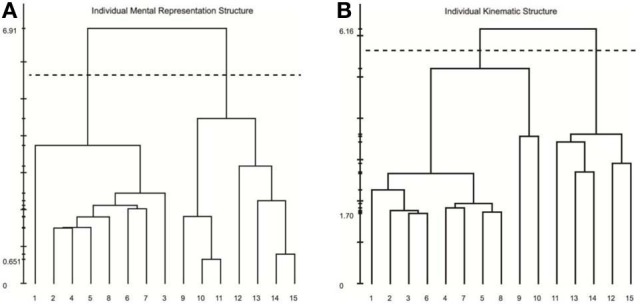
**Individual dendrograms from one participant for the mental representation (A) and swing kinematics (B)**. The numbers on the horizontal axis relate to the concept number, the numbers on the vertical axis display Euclidean distances. The lower the link between related concepts, the lower is the Euclidean distance between the corresponding concepts in feature space. The horizontal dotted line marks *d*_*crit*_ value for a given α-level (*d*_*crit*_ = 5.64; α = 0.001): links below this line are considered statistically relevant. Concepts: (1) head, (2) chest, (3) left shoulder, (4) left elbow, (5) left hand, (6) right shoulder, (7) right elbow, (8) right hand, (9) hips, (10) left thigh, (11) left knee, (12) left foot, (13) right thigh, (14) right knee, (15) right foot.

Results of our analyses indicated a high degree of consistency and similarity between the structure of mental representations and movement kinematics. Examination of group mean dendrograms for both the mental representation structure and the major movement kinematic structure revealed a significant similarity between the two structures (λ = 0.71; λ_*crit*_ = 0.68; for more details, see Schack, 2012). That is to say that the structure revealed in the memory representation was statistically equivalent to the movement structure revealed by the STKD procedure (see Figure 5). Specifically, both the representation structure and kinematic structure displayed two distinct clusters representing the upper and lower body (*p* < 0.001). However, one difference emerged between the two structures in regard to the body segment “hips,” such that in the mental representation, the hips were coupled with the functional relevance of the upper body, whereas the kinematic structure indicated that the hips were more closely coupled to the movement of the lower body. In this case, a mismatch exists between the group mental and group kinematic structure. Additionally, a comparison of the mental and kinematic structures on an individual level^3^ revealed that 5 out of 9 individuals displayed significantly similar structures (*M*_λ_ = 0.63, *SD* = 0.09; λ_*crit*_ = 0.68, *n* = 9). Figure 6 displays the similarity in the mental and kinematic structures for a single participant whose structures are statistically invariant (λ = 0.71; λ_*crit*_ = 0.68). Interestingly, the degree of task experience was not related to the degree of similarity between representation and kinematic structure in our current sample (*r* = −0.385, *p* = 0.31). However, future research is needed to determine why some individuals display stronger connections between mental and kinematic structures. To this extent, examination of differences between intact groups of novices and experts may provide further clarity into this connection.

In addition to the structural invariance measures based on the cluster solutions, significant correlations were evident between the mental and kinematic distance matrices. These correlations represent the degree of similarity between the coupling of body segments in feature space, as defined by the SDA-M and STKD procedures. Specifically, the group mean mental and kinematic distance matrices indicated a strong and positive correlation (*r* = 0.629, *p* < 0.001). As such, there is a close relationship between the relative body segments in both memory and physical execution. Likewise, significant correlations were evident on an individual level. For all participants, the correlation between the mental and kinematic distance matrices indicated significant positive correlations with values ranging from *r* = 0.242 to *r* = 0.712. Noteworthy, the correlations between mental and physical distance matrices remained remarkably stable over repeated trials within each individual, with an average correlation standard deviation of 0.02 across all participants.

**Figure 7:**
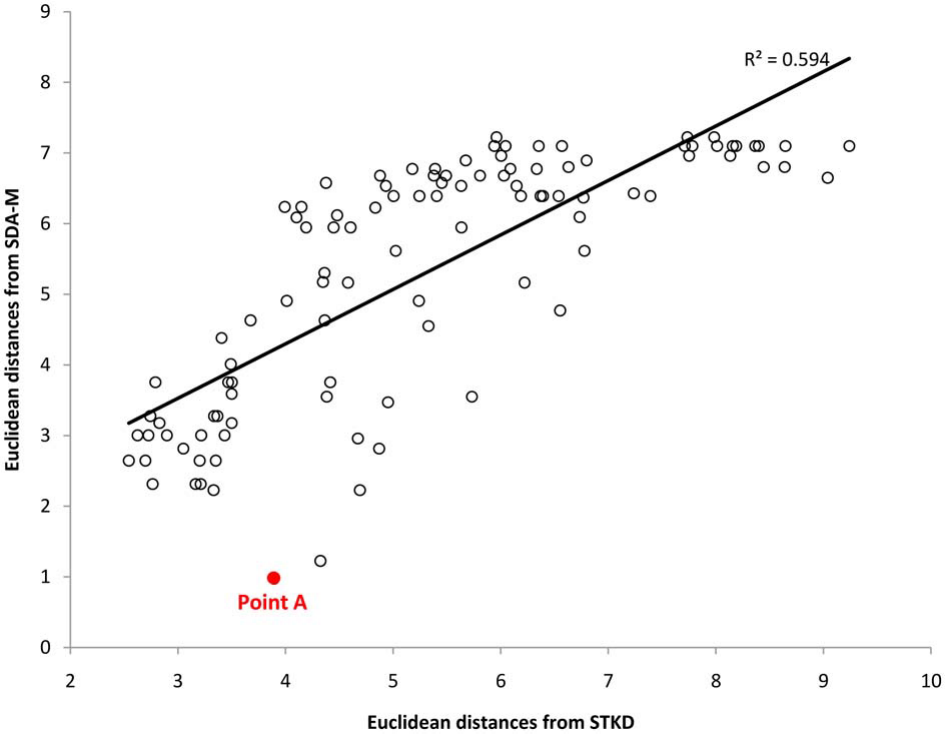
**Scatter plot representing the relation between the distance matrices derived from the SDA-M and STKD procedures for one participant**. Point A represents an outlier and corresponds to the relationship between the left thigh and hip movement, thus indicating a mismatch between cognitive representation and the major kinematic feature of movement.

Examining the scatter plot of the relationship between the mental and kinematic distance matrices also provides important insights into the link between representation and movement. Specifically, examination of outliers can be used as a diagnostic procedure to identify mismatches between an individual's movement representation and the physical execution of the movement. For instance, Figure 7 displays the relationship between the mental and kinematic distance matrices for one participant. As can be seen, the points largely lay along the trend line, representing a strong relationship; however, point A represents a strong deviation from the predicted relationship. Examination of point A reveals that the left knee and left thigh were not similarly coupled in the execution of the movement like they were in the mental representation. This difference corresponds with the differences observed in the structural analyses as seen in Figure 6 from the same participant. Such differences may suggest areas for targeted skill interventions by coaches, trainers, or physical therapists. Future research is needed to determine the qualitative impact on motor performance where such mismatches between mental and kinematic structure exist.

### Discussion of findings

The above findings further confirm the close link between mental representations and the execution of motor actions. These results build on previous findings that have found functional links between representational chunks and specific biomechanical parameters within a given movement (e.g., Schack, 2003; Schütz et al., 2009). Specifically, our findings suggest that mental representations not only play a key role in guiding key biomechanical parameters of a movement, but also guide the overall structure of the action. To this end, we observed a close relationship between the overall structure of both the mental representation and movement kinematics on both a group and individual level.

The close link between representation and kinematic structure is consistent with the Cognitive Architecture model proposed by Schack (2004), which suggests that motor skills are organized within hierarchical memory structures comprised of elementary components or transitional states of complex movements. These memory structures act as a cognitive reference for the unfolding of action, such that they serve to govern the tuning of motor commands and muscular activity patterns. As such, a tight correlation is predicted to exist between the structure of mental representation and the structure of movement.

As we show, the structures observed within the movement kinematics are reflected within the mental representations of the participants. Interestingly, the degree of association or invariance between the mental representation and the movement structure did not appear to be dependent on the level of skill of the individual. Although our sample consisted of relatively few participants, thus likely making any relationship difficult to observe, the lack of a moderating effect of skill level is not entirely baseless. While research has shown that experts have more elaborate and hierarchically-organized mental representations (e.g., Bläsing et al., 2009), the overall function of the representation is to guide the unfolding of motor patterns. As such, regardless of the quality of the representation, the movement unfolds in a manner that is consistent with the representation structure, and thus movements and representations would be clearly correlated across all skill levels. However, this assumption warrants future research to confirm or reject the influence of skill level and other factors on the degree of association between movement and representation structure.

Examining the structural relatedness between mental representations and movements has a number of practical applications. As has been demonstrated, investigating the mismatch between memory structures and kinematic synergies may be useful for diagnosing movement disorders or guiding training strategies. To this extent, investigation of mental representations via the SDA-M method has successfully been shown to identify representational problems within stroke patients who display movement deficiencies (Braun et al., 2007). Similarly, incongruence between mental and kinematic structures may be a key factor in determining how deficient mental representations are manifested within the overall motor production. Additionally, the degree of invariance between mental and kinematic structures may act as a useful benchmark for examining the efficacy of artificial cognitive systems within modern robotics. As robotic platforms make qualitative leaps in the areas of perceptual, cognitive, and motor capabilities, cognitive architectures designed to effectively integrate these functions become essential. The field of cognitive robotics is increasingly turning to biological models of action organization to guide the development of efficient and natural cognitive control systems (e.g., Schack and Ritter, 2009, in press; Maycock et al., 2010). In this direction, first steps have already been taken in modeling higher level cognitive representations derived from human data into robotic grasping applications (Maycock et al., 2010). The extent to which artificial cognitive systems efficiently represent and guide complex actions may be distinguishable based upon our proposed method, which would indicate a level of cognitive sophistication similar to that of its biological counterparts.

## Summary

The present article reviewed the substantial work on mental representations underlying complex action in humans. In doing so, we proposed a new experimental approach to capture the relationship between mental representation and the kinematic structure of movement. The STKD method presented in this paper allows for the segmentation of any recorded movement into a minimal number of independent spatial-temporal features. This method has been found to effectively elicit the hierarchically-organized key kinematic elements of a movement in different spatio-temporal scales. Based on these analyses, we presented a first step toward linking the memory structure of a complex motor task to the unfolding movement dynamics. Results from our analyses indicated a clear structural relationship between the motor representation in long-term memory and the functional structure of movement kinematics. These findings support the theoretical perspective that complex actions are planned and performed with the help of structured cognitive representations in long-term memory that act to guide the biomechanical organization of movements (Hommel et al., 2001; Mechsner et al., 2001; Schack and Mechsner, 2006; Hoffmann et al., 2007). Implications of these findings are important for a number of movement related domains, including physical therapy, sports training, and artificial cognitive systems. While the present paper presents an important first step in the direction of linking cognitive and biomechanical structures, much work remains to extend the current findings to new task domains while also exploring the variables that moderate this relationship.

### Conflict of interest statement

The authors declare that the research was conducted in the absence of any commercial or financial relationships that could be construed as a potential conflict of interest.

## Footnotes

^1^The body parts used as concepts within the SDA-M procedure, and subsequently tracked via motion capture, consisted of 1) head, 2) chest, 3) left shoulder, 4) left elbow, 5) left hand, 6) right shoulder, 7) right elbow, 8) right hand, 9) hips, 10) left thigh, 11) left knee, 12) left foot, 13) right thigh, 14) right knee, and 15) right foot.

^2^For more details on the SDA-M analysis, please see Schack (2012).

^3^For the individual comparisons, we utilized the SDA-M results and the STKD results from a single kinematic trial.
